# Breast reconstruction-related complications from postmastectomy radiation therapy in stage II–III breast cancer: sub-analysis of a multi-institutional observational study (Reborn-03)

**DOI:** 10.1007/s12282-025-01799-z

**Published:** 2025-11-18

**Authors:** Shinsuke Sasada, Wakako Tsuji, Noriyuki Watanabe, Ayaka Shimo, Natsue Uehiro, Takahiro Tsukioki, Naomi Nagura, Shoichi Tomita, Hiroko Nogi, Kei Yamaguchi, Kazuhiko Yamagami, Akiko Ogiya, Hiromi Suetsugu, Yuki Nakamura, Chikako Yamauchi, Hirohito Seki

**Affiliations:** 1 Department of Surgical Oncology, Research Institute for Radiation Biology and Medicine, Hiroshima University, 1-2-3 Kasumi, Minami-ku, Hiroshima City, Hiroshima 734-8551 Japan; 2 Department of Breast Surgery, Shiga General Hospital, Moriyama, Shiga Japan; 3 Department of Breast and Endocrine Surgery, Osaka International Cancer Institute, Osaka, Japan; 4 Department of Breast and Endocrine Surgery, St. Marianna University School of Medicine, Kawasaki, Kanagawa Japan; 5 Department of Breast Surgical Oncology, Breast Oncology Center, Cancer Institute Hospital, Japanese Foundation for Cancer Research, Tokyo, Japan; 6 Department of Breast and Endocrine Surgery, Okayama University Hospital, Okayama, Japan; 7 Department of Breast Surgical Oncology, St. Luke’s International Hospital, Tokyo, Japan; 8 Department of Plastic and Reconstructive Surgery, Tokyo Metropolitan Cancer and Infectious Diseases Center, Komagome Hospital, Tokyo, Japan; 9 Department of Breast and Endocrine Surgery, Jikei University School of Medicine, Tokyo, Japan; 10 Department of Breast Surgery, Saitama Medical Center, Saitama, Japan; 11 Department of Breast Surgery, Shinko Hospital, Kobe, Hyogo Japan; 12 Department of Breast Surgery, Japanese Red Cross Medical Center, Tokyo, Japan; 13 Department of Breast Surgery, Kyoto City Hospital, Kyoto, Japan; 14 Department of Breast Surgery, Kyoto University Hospital, Kyoto, Japan; 15 Department of Breast Surgery, Kyorin University School of Medicine, Tokyo, Japan

**Keywords:** Breast cancer, Breast reconstruction, Mastectomy, Postmastectomy radiation therapy, Postoperative complication

## Abstract

**Background:**

Although postmastectomy radiation therapy (PMRT) is a standard treatment for high-risk breast cancer, it adds the risk of complications after breast reconstruction (BR).

**Methods:**

This multi-institutional cohort study included patients with stage II–III breast cancer—defined as tumor size ≥ 5 cm, lymph node involvement, and/or skin/chest wall invasion—who underwent immediate or delayed BR after mastectomy between January 2008 and December 2018. We retrospectively investigated the relationship between PMRT and BR-related complications after adjusting for patient characteristics and BR method.

**Results:**

Among 1138 patients (1101 immediate and 37 delayed BR), 427 (37.5%) underwent PMRT. The cohort included 238 (20.9%) patients with tumors ≥ 5 cm, 725 (63.7%) with 1–3 lymph node metastases, and 257 (22.6%) with ≥ 4. BR methods included 750 prosthetic, 385 autologous, and 3 fat graft procedures. The overall complication rates were 25.2% and 31.5% in the non-PMRT and PMRT groups, respectively (*p* = 0.028). In prosthetic BR, capsule contracture (*p* < 0.001) and infection (*p* = 0.014) were more frequent in the PMRT than the non-PMRT group, whereas no significant difference was observed in complication rates between two groups in autologous BR. Multivariate analysis identified PMRT as an independent risk factor for BR-related complications (odds ratio, 1.47; *p* = 0.032). The reoperation rate was similar in two groups.

**Conclusions:**

PMRT was associated with an increased incidence of BR-related complications in prosthetic BR, but not with reoperation, irrespective of the reconstruction method.

**Supplementary Information:**

The online version contains supplementary material available at 10.1007/s12282-025-01799-z.

## Introduction

Breast cancer is the most prevalent malignancy among women worldwide, and the same is true in Japan [1, 2]. Mastectomy remains the primary surgery performed in the treatment of breast cancer, and is now performed more frequently than breast-conserving surgery, with breast reconstruction (BR) performed in approximately 10% of mastectomy cases [3–5]. Postmastectomy BR restores breast symmetry and offers high levels of both patient satisfaction and health-related quality of life, albeit these benefits are associated with the risk of postoperative complications [6, 7].

Postmastectomy radiation therapy (PMRT) is recommended for patients with T3–4 tumors, positive nodes, or involved resection margins to reduce locoregional recurrence and breast cancer mortality, even if patients have previously undergone BR [8–11]. However, PMRT increases the risk of BR-related complications and reduces patient satisfaction, particularly among patients who have undergone prosthetic BR [12–14]. Previous studies reported that postoperative complications delayed the initiation of systemic therapy, although it was unclear whether they worsened overall patient prognoses [15, 16]. As the Japan Oncoplastic Breast Surgery Society (JOPBS) does not recommend the use of breast prostheses in patients with high-risk breast cancer who require PMRT, no systematic data are available in Japan regarding the impact of PMRT on BR-related complications [17].

This multicenter study, therefore, investigated the relationship between PMRT and BR-related complications in patients with stage II–III breast cancer.

## Materials and methods

### Patients

This retrospective study, conducted as a part of a JOPBS collaborative study, reviewed the medical data of patients with stage II–III breast cancer who underwent mastectomy and immediate or delayed BR at 15 institutes between January 2008 and December 2018. Eligibility criteria were defined as a tumor size ≥ 5 cm, lymph node involvement, and/or skin/chest wall invasion. Patients with stage IV breast cancer, synchronous and/or metachronous bilateral breast cancer, or previous radiation therapy after breast-conserving surgery for ipsilateral breast cancer were excluded. Of the 1143 eligible patients, 5 were excluded from the analyses due to missing PMRT-related information.

### Complications related to BR

Complications were classified based on the Clavien–Dindo scale as follows: hemorrhage; seroma; flap necrosis; nipple necrosis and infection; capsule contracture (Baker classification grade III–IV) [18], rupture, malposition, or loss of reconstruction material; and reoperation. The frequency of nipple necrosis was calculated in patients who underwent nipple-sparing mastectomy while capsule contracture, rupture, malposition, and loss of reconstructive material were observed in patients who underwent prosthetic BR.

### Statistical analysis

Baseline characteristics are presented as numbers and percentages. Statistical comparisons of categorical variables were performed using the Chi-squared test, whereas continuous variables were compared using the Mann–Whitney U test. Risk factors for complications were evaluated through logistic regression analyses, and odds ratios and confidence intervals were estimated. Differences were considered statistically significant at two-tailed *p*-values < 0.05.

## Results

Patient characteristics are presented in Table 1. Among the 1138 patients included in this analysis, 427 (37.5%) underwent PMRT. Compared with patients who did not undergo PMRT, those who did were younger, had larger tumors and more nodal metastases, and more frequently underwent skin-sparing mastectomies, axillary dissections, and treatment with chemotherapy. BR methods included 750 breast prostheses, 385 autologous tissues, and 3 fat grafts. Thirty-seven (3.3%) patients underwent a delayed BR. Delayed BR was completed at a median of 23.8 months (interquartile range [IQR] 13.5–29.5) following the initial surgery, and at 20.8 months (IQR 10.2–25.7) following PMRT, with a median follow-up period of 98.2 months (IQR 69.3–121.7).

**Table 1:** Patient characteristics

	Total	non-PMRT	PMRT	*p*
	(*n* = 1138)	(*n* = 711)	(*n* = 427)	
Age (y), median (range)	46 (23–76)	46 (23–76)	45 (24–71)	0.008
Body mass index				0.671
<25	947 (83.2)	587 (82.8)	360 (84.7)	
25–30	165 (14.5)	107 (15.1)	58 (13.6)	
30≤	22 (1.9)	15 (2.1)	7 (1.6)	
Unknown	4 (0.4)			
Smoking				0.747
Never	842 (74.0)	513 (76.9)	329 (78.9)	
Former	118 (10.4)	75 (11.2)	43 (10.3)	
Current	124 (10.9)	79 (11.8)	45 (10.8)	
Unknown	54 (4.7)			
pT				< 0.001
0	34 (3.0)	25 (3.5)	9 (2.1)	
1	432 (38.0)	325 (45.7)	107 (25.1)	
2	405 (35.6)	226 (31.8)	179 (41.9)	
3	238 (20.9)	110 (15.5)	128 (30.0)	
Unknown	29 (2.5)	25 (3.5)	4 (0.9)	
Nodal metastasis				< 0.001
0	156 (13.7)	127 (17.9)	29 (6.8)	
1–3	725 (63.7)	547 (76.9)	178 (41.7)	
4≤	257 (22.6)	37 (5.2)	220 (51.5)	
Subtype				0.148
Luminal	861 (75.7)	532 (74.8)	329 (77.0)	
HER2	202 (17.8)	135 (19.0)	67 (15.7)	
Triple-negative	71 (6.2)	40 (5.6)	31 (7.3)	
Unknown	4 (0.3)	4 (0.6)	0 (0)	
Breast surgery				0.009
Mastectomy	474 (41.6)	314 (44.2)	160 (37.5)	
Skin-sparing mastectomy	348 (30.6)	195 (27.4)	153 (35.8)	
Nipple-sparing mastectomy	316 (27.8)	202 (28.4)	114 (26.7)	
Axillary surgery				< 0.001
Sentinel lymph node biopsy	282 (24.8)	225 (31.6)	57 (13.3)	
Axillary dissection	856 (75.2)	486 (68.4)	370 (86.7)	
Breast reconstruction				0.111
Immediate	1101 (96.7)	693 (97.5)	408 (95.6)	
Delayed	37 (3.3)	18 (2.5)	19 (4.4)	
Reconstruction material				0.075
Breast prosthesis	750 (65.9)	482 (67.8)	268 (62.8)	
Autologous tissue	385 (33.8)	226 (31.8)	159 (37.2)	
Fat graft	3 (0.3)	3 (0.4)	0 (0)	
Chemotherapy				
Neoadjuvant	322 (28.3)	177 (24.9)	145 (34.0)	0.001
Adjuvant	633 (55.6)	337 (47.5)	296 (69.3)	< 0.001

*HER2* human epidermal growth factor receptor 2, *PMRT* postmastectomy radiation therapy

### Complications related to BR

Details of the documented BR-related complications are shown in Table 2. Complications occurred more frequently in the PMRT group than in the non-PMRT group (31.5 vs. 25.2%, respectively; *p* = 0.028), as did capsule contractures (46.5 vs. 18.4%, respectively; *p* < 0.001), although there was no significant difference in grade 3 or higher complications between the two groups. Loss of BR materials and reoperation occurred in 8.2 and 11.7% of the PMRT group and 8.0 and 9.3% of the non-PMRT group, respectively. Among the 37 patients who underwent delayed BR, 18 (48.6%) received PMRT, and 21 (56.8%) underwent autologous BR. Only one patient required reoperation owing to grade 3 hemorrhage.

**Table 2:** Complications related to breast reconstruction based on postmastectomy radiation therapy

	non-PMRT (*n* = 711)		PMRT (*n* = 427)		*p*	
	Any grade	≥ Grade 3	Any grade	≥ Grade 3	Any grade	≥ Grade 3
All	179 (25.2)	61 (8.6)	134 (31.5)	41 (9.6)	0.028	0.635
Hemorrhage	22 (3.1)	3 (0.4)	12 (2.8)	2 (0.5)	0.925	1
Seroma	13 (1.8)	2 (0.3)	12 (2.8)	1 (0.2)	0.377	1
Flap necrosis	45 (6.3)	20 (2.8)	38 (8.9)	13 (3.1)	0.135	0.967
Nipple necrosis^†^	34 (16.7)	2 (1.0)	22 (19.1)	1 (0.9)	0.688	1
Infection	31 (4.4)	22 (3.1)	29 (6.8)	19 (4.5)	0.101	0.307
Capsule contracture^#^	45 (18.4)	–	53 (46.5)	–	< 0.001	–
Rapture	6 (1.2)	–	2 (0.7)	–	0.790	–
Malposition	5 (1.0)	–	5 (1.9)	–	0.538	–
Loss of reconstruction materials	–	39 (8.0)	–	22 (8.2)	–	1
Reoperation	–	66 (9.3)	–	50 (11.7)	–	0.227

Calculated excluding cases with unknown events

*PMRT* postmastectomy radiation therapy

^†^Including only patients underwent nipple-sparing mastectomy

^#^ Including only patients with grade III–IV by Baker classification

Complications associated with PMRT and each BR method are presented in Table 3. PMRT implementation rates were 35.4% (268/758) in patients who underwent prosthetic BR and 41.1% (158/384) in those who underwent autologous BR. Overall, complications were more common in the PMRT group than in the non-PMRT group among patients who underwent prosthetic BR (36.2 vs. 27.3%, respectively; *p* = 0.014), as were infection (9.7 vs. 4.8%, respectively; *p* = 0.014) and capsule contracture (50.0 vs. 19.0%, respectively; *p* < 0.001), whereas there was no significant difference among those who underwent autologous BR.

**Table 3:** Complications related to breast reconstruction based on postmastectomy radiation therapy and reconstructive method

	Prosthetic			Autologous		
	Non-PMRT	PMRT	*p*	Non-PMRT	PMRT	*p*
All	131 (27.3)	97 (36.2)	0.014	48 (21.2)	37 (23.4)	0.703
Hemorrhage	15 (3.1)	9 (3.4)	1	7 (3.1)	3 (1.9)	0.689
Seroma	8 (1.7)	6 (2.2)	0.785	5 (2.2)	6 (3.8)	0.545
Flap necrosis	29 (6.0)	23 (8.6)	0.246	16 (7.1)	15 (9.5)	0.507
Nipple necrosis^†^	26 (17.2)	11 (14.5)	0.735	8 (16.0)	11 (28.2)	0.257
Infection	23 (4.8)	26 (9.7)	0.014	8 (3.5)	3 (1.9)	0.524
Capsule contracture^#^	44 (19.0)	53 (50.0)	< 0.001	–	–	–
Rapture	6 (1.2)	2 (0.7)	0.790	–	–	–
Malposition	5 (1.0)	5 (1.9)	0.538	–	–	–
Loss of reconstruction materials	36 (7.5)	21 (7.8)	0.970	–	–	–
Reoperation	53 (11.0)	36 (13.4)	0.383	13 (5.8)	14 (8.8)	0.341

Calculated excluding cases with unknown events

*PMRT* postmastectomy radiation therapy

^†^Including only patients underwent nipple-sparing mastectomy

^#^ Including only patients with grade III–IV by Baker classification

### Impact of PMRT on BR-related complications

In the univariate analysis, obesity (body mass index ≥ 25), nipple-sparing mastectomy, immediate BR, prosthetic BR, pT stage ≤ 2, nodal metastasis, adjuvant chemotherapy, and PMRT were related to complications. In the multivariate analysis, PMRT was an independent risk factor for complications (odds ratio, 1.47; 95% confidence interval [CI], 1.03–2.10: *p* = 0.032), as were postmenopausal status, obesity, nipple-sparing mastectomy, prosthetic BR, and nodal metastasis (Table 4). PMRT was significantly associated with complications in patients who underwent prosthetic BR (odds ratio, 2.01; 95% CI, 1.29–3.13; *p* = 0.002), although the same was not true for those who underwent autologous BR (odds ratio, 0.78; 95% CI, 0.42–1.47; *p* = 0.444) (Supplementary Tables S1, S2). PMRT did not, however, increase patients’ risk of reoperation (Supplementary Table S3).

**Table 4:** Logistic regression analysis for complications related to breast reconstruction

Factors	Univariate analysis		Multivariate analysis	
	OR (95% CI)	*P*	OR (95% CI)	*P*
Postmenopause	1.16 (0.85–1.57)	0.346	1.41 (1.00–1.97)	0.047
Body mass index ≥ 25	1.38 (0.98–1.93)	0.063	1.52 (1.05–2.18)	0.025
Smoking				
Never	Reference		Reference	
Former	1.25 (0.82–1.89)	0.304	1.36 (0.87–2.12)	0.178
Current	1.40 (0.93–2.09)	0.104	1.28 (0.83–1.97)	0.269
Breast surgery				
Mastectomy	Reference		Reference	
Skin-sparing mastectomy	0.92 (0.66–1.28)	0.621	0.93 (0.66–1.32)	0.691
Nipple-sparing mastectomy	2.02 (1.48–2.76)	< 0.001	2.45 (1.73–3.46)	< 0.001
Axillary surgery				
Sentinel lymph node biopsy	Reference		Reference	
Axillary dissection	0.79 (0.59–10.6)	0.116	0.67 (0.47–0.95)	0.025
Breast reconstruction				
Immediate	Reference		Reference	
Delayed	0.15 (0.04–0.65)	0.011	0.25 (0.06–1.09)	0.066
Reconstruction material				
Breast prosthesis	Reference		Reference	
Autologous tissue	0.64 (0.49–0.86)	0.003	0.69 (0.50–0.94)	0.024
pT stage ≥ 3	0.70 (0.50–0.98)	0.040	0.75 (0.51–1.11)	0.152
Nodal metastasis				
0	Reference		Reference	
1–3	2.56 (1.59–4.13)	< 0.001	2.55 (1.44–4.50)	0.001
4≤	2.62 (1.55–4.43)	< 0.001	2.50 (1.30–4.82)	0.006
Neoadjuvant chemotherapy	0.74 (0.55–1.00)	0.050	0.92 (0.61–1.39)	0.797
Adjuvant chemotherapy	1.45 (1.11–1.90)	0.006	1.12 (0.79–1.60)	0.520
Postmastectomy radiation therapy	1.36 (1.04–1.77)	0.024	1.47 (1.03–2.10)	0.032

*CI* confidence interval, *OR* odds ratio

## Discussion

Our analysis revealed the impact of PMRT on BR-related complications in patients with stage II–III breast cancer who underwent mastectomy and BR as part of the Reborn-03 study. We found that PMRT was associated with more frequent postoperative complications, especially among patients who underwent prosthetic BR; however, it did not pose a risk for severe complications or reoperation.

Postoperative complications are associated with a variety of patient- and treatment-related factors [19]. Patient-related factors include advanced age, obesity, smoking, and hypertension. In the present study, postmenopausal status and increased body mass index were risk factors, although smoking was not. The reason for this discrepancy might be that only 21.3% of the patients were smokers, the amount of smoking was unknown, and participants were limited to high-risk individuals. Nipple necrosis, a unique complication of nipple-sparing mastectomy, is a treatment-related factor [19]. Although delayed BR is no safer than immediate BR, there were too few patients with delayed BR to be evaluated in this study [20]. The main difference between BR in Japan and other countries is that the breast prosthesis is placed under the pectoralis major muscle, and the acellular dermal matrix (ADM) is not approved. The safety of prepectoral reconstruction and ADM have been documented, and our findings on post-BR complications are compatible with those in other countries [21, 22]. Additionally, neoadjuvant chemotherapy does not seem to increase BR-related complications [23, 24]. In the present study, axillary dissection and nodal metastasis were inversely associated with complications; however, the reasons for this inverse association were unclear from our findings, meaning confounding factors may not have been adequately excluded.

PMRT is a significant risk factor for BR-related complications; however, its impact depends on the reconstruction method [13, 14]. Patients who underwent prosthetic BR experienced more complications in the PMRT group; however, there was no significant difference between the two groups among patients who underwent autologous BR. Even among patients who underwent prosthetic BR, capsular contracture was the most common complication associated with PMRT, while loss of reconstruction materials and reoperation rates did not increase. Capsular contracture represents a well-documented complication associated with breast implant placement. Following implantation, the host tissue typically initiates a foreign body response, leading to the development of a fibrous capsule surrounding the implant. This is influenced by factors such as the type of reconstructive material used, infection, and hematoma formation [25]. Furthermore, radiation-induced fibrosis constitutes a significant etiological factor; radiation exposure, which can induce fibrotic changes in the soft tissue envelope and underlying musculature [26]. In cases requiring PMRT, autologous BR is the preferred method, although prosthetic BR may also be an option, depending on patient preferences.

The importance of patient-reported outcomes (PROs) has recently been posited as a quality of life (QOL) assessment in patients with breast cancer; however, the impact of postoperative complications on QOL remains unclear. A single-institution cross-sectional survey reported that post-operative complications did not negatively impact QOL [27], whereas another study reported that flap necrosis after nipple-sparing mastectomy was related to lower short-term QOL, which returned to baseline by 1 year [28]. In the PRO study of patients from the JOPBS collaborative study, most of whom had undergone reconstruction more than three years ago, PMRT was associated with a lower QOL [29]. Autologous BR appears to provide superior patient-reported QOL compared with prosthetic BR among patients receiving PMRT [13]. Even for patients planning for PMRT, BR using an appropriate method should not be abandoned, considering the patient’s background and preferences.

This study has some limitations worth mentioning. First, an inherent selection bias was associated with the retrospective study design; therefore, incomprehensible risk factors remained after the multivariate adjustment. While most BR is performed in patients with early stage breast cancer, the inclusion criterion for high-risk diseases is a strength of this study. Second, there was insufficient information on complications among the included patients. Most mild complications, such as the Clavien–Dindo classification grade 1 or Baker grade I–II, and the timing of complication onset, were missing. Given that infections most commonly occur during the early postoperative phase preceding PMRT, the existence of a direct causal association with PMRT remains uncertain. Third, late complications were unknown. The median follow-up period in this study was 8 years; therefore, it is thought that further observations will reveal more cases of implant rupture, replacement, and/or removal.

In conclusion, we clarified the relationship between PMRT and BR-related complications in patients with stage II–III breast cancer in a large Japanese cohort. PMRT appeared to increase the capsular contracture in patients who have undergone prosthetic BRs; however, it did not affect the reoperation rate. From a safety prospective, autologous BR is the preferred option for patients requiring PMRT, although prosthetic BR should also be considered for selected patients through shared decision-making that reflects patient preferences.

## Supplementary Information

Below is the link to the electronic supplementary material.

Supplementary Material 1

## Data Availability

The datasets generated and/or analyzed during the present study are not publicly available due to license restrictions. Data are available from the corresponding author upon reasonable request.
