# Supplementary material for: Breast reconstruction-related complications from postmastectomy radiation therapy in stage II–III breast cancer: sub-analysis of a multi-institutional observational study (Reborn-03)
Source: Breast Cancer. 2025 Nov 18;33(1):243–50. doi: 10.1007/s12282-025-01799-z (PMC12789235; doi:10.1007/s12282-025-01799-z)
Supplement: Supplementary file 1 — Supplementary Material 1 [file 12282_2025_1799_MOESM1_ESM.docx]

Supplementary table S1. Logistic regression analysis of complications among patients who underwent prosthetic breast reconstruction.

| Factors | Univariate analysis | | Multivariate analysis | |
| --- | --- | --- | --- | --- |
|  | OR (95% CI) | *p* | OR (95% CI) | *p* |
| Postmenopause | 1.30 (0.89–1.89) | 0.173 | 1.52 (1.00–2.30) | 0.050 |
| Body mass index ≥25 | 1.42 (0.94–2.14) | 0.096 | 1.42 (0.91–2.22) | 0.121 |
| Smoking |  |  |  |  |
| Never | Reference |  | Reference |  |
| Former | 1.19 (0.71–1.99) | 0.513 | 1.29 (0.75–2.23) | 0.362 |
| Current | 1.63 (1.03–2.57) | 0.037 | 1.49 (0.91–2.43) | 0.115 |
| Breast surgery |  |  |  |  |
| Mastectomy | Reference |  | Reference |  |
| Skin-sparing mastectomy | 0.66 (0.45–0.98) | 0.040 | 0.72 (0.47–1.10) | 0.130 |
| Nipple-sparing mastectomy | 1.43 (0.99–2.06) | 0.058 | 1.89 (1.24–2.87) | 0.003 |
| Axillary surgery |  |  |  |  |
| Sentinel lymph node biopsy | Reference |  | Reference |  |
| Axillary dissection | 0.85 (0.61–1.19) | 0.354 | 0.67 (0.44–1.00) | 0.052 |
| Breast reconstruction |  |  |  |  |
| Immediate | Reference |  | Reference |  |
| Delayed | 0.38 (0.08–1.69) | 0.201 | 0.65 (0.12–3.52) | 0.620 |
| pT stage ≥3 | 0.57 (0.38–0.85) | 0.006 | 0.65 (0.41–1.03) | 0.064 |
| Nodal metastasis |  |  |  |  |
| 0 | Reference |  | Reference |  |
| 1–3 | 3.30 (1.79–6.10) | <0.001 | 2.72 (1.32–5.59) | 0.006 |
| 4≤ | 3.04 (1.56–5.93) | 0.001 | 2.16 (0.94–4.97) | 0.069 |
| Neoadjuvant chemotherapy | 0.71 (0.48–1.03) | 0.074 | 0.76 (0.45–1.28) | 0.307 |
| Adjuvant chemotherapy | 1.47 (1.07–2.03) | 0.018 | 1.01 (0.66–1.54) | 0.981 |
| Postmastectomy radiation therapy | 1.51 (1.10–2.08) | 0.014 | 2.01 (1.29–3.13) | 0.002 |

CI, confidence interval; OR, odds ratio.

Supplementary table S2. Logistic regression analysis of complications among patients who underwent autologous breast reconstruction.

| Factors | Univariate analysis | | Multivariate analysis | |
| --- | --- | --- | --- | --- |
|  | OR (95% CI) | *p* | OR (95% CI) | *p* |
| Postmenopause | 1.02 (0.59–1.75) | 0.955 | 1.27 (0.70–2.32) | 0.429 |
| Body mass index ≥25 | 1.37 (0.75–2.51) | 0.306 | 1.97 (0.98–3.95) | 0.057 |
| Smoking |  |  |  |  |
| Never | Reference |  | Reference |  |
| Former | 1.38 (0.67–2.84) | 0.376 | 1.44 (0.66–3.14) | 0.366 |
| Current | 0.64 (0.24–1.72) | 0.374 | 0.70 (0.24–2.02) | 0.510 |
| Breast surgery |  |  |  |  |
| Mastectomy | Reference |  | Reference |  |
| Skin-sparing mastectomy | 1.77 (0.96–3.27) | 0.068 | 1.71 (0.89–3.29) | 0.108 |
| Nipple-sparing mastectomy | 4.30 (2.37–7.82) | <0.001 | 4.63 (2.39–8.96) | <0.001 |
| Axillary surgery |  |  |  |  |
| Sentinel lymph node biopsy | Reference |  | Reference |  |
| Axillary dissection | 0.83 (0.44–1.57) | 0.561 | 0.73 (0.35–1.56) | 0.418 |
| Breast reconstruction |  |  |  |  |
| Immediate | Reference |  | Reference |  |
| Delayed | 0.00 (0.00–Inf) | 0.978 | 0.00 (0.00–Inf) | 0.987 |
| pT stage ≥3 | 1.11 (0.60–2.07) | 0.739 | 1.30 (0.61–2.77) | 0.504 |
| Nodal metastasis |  |  |  |  |
| 0 | Reference |  | Reference |  |
| 1–3 | 1.54 (0.71–3.34) | 0.272 | 2.48 (0.91–6.77) | 0.076 |
| 4≤ | 1.99 (0.84–4.68) | 0.116 | 3.82 (1.21–12.1) | 0.022 |
| Neoadjuvant chemotherapy | 0.94 (0.57–1.55) | 0.800 | 1.16 (0.57–2.39) | 0.680 |
| Adjuvant chemotherapy | 1.30 (0.80–2.11) | 0.292 | 1.24 (0.63–2.44) | 0.543 |
| Postmastectomy radiation therapy | 1.13 (0.70–1.85) | 0.613 | 0.78 (0.42–1.47) | 0.444 |

CI, confidence interval; OR, odds ratio.

Supplementary table S3. Logistic regression analysis for reoperation.

| Factors | Univariate analysis | | Multivariate analysis | |
| --- | --- | --- | --- | --- |
|  | OR (95% CI) | *p* | OR (95% CI) | *p* |
| Postmenopause | 1.49 (0.97–2.29) | 0.067 | 1.61 (1.02–2.53) | 0.398 |
| Body mass index ≥25 | 1.74 (1.10–2.75) | 0.018 | 1.82 (1.13–2.93) | 0.013 |
| Smoking |  |  |  |  |
| Never | Reference |  | Reference |  |
| Former | 1.05 (0.57–1.94) | 0.883 | 1.12 (0.59–2.10) | 0.734 |
| Current | 0.91 (0.48–1.71) | 0.762 | 0.89 (0.46–1.70) | 0.719 |
| Breast surgery |  |  |  |  |
| Mastectomy | Reference |  | Reference |  |
| Skin-sparing mastectomy | 0.91 (0.57–1.45) | 0.686 | 0.92 (0.56–1.49) | 0.721 |
| Nipple-sparing mastectomy | 1.05 (0.66–1.66) | 0.85 | 1.21 (0.73–2.00) | 0.463 |
| Axillary surgery |  |  |  |  |
| Sentinel lymph node biopsy | Reference |  | Reference |  |
| Axillary dissection | 0.81 (0.53–1.24) | 0.335 | 0.73 (0.45–1.21) | 0.223 |
| Breast reconstruction |  |  |  |  |
| Immediate | Reference |  | Reference |  |
| Delayed | 0.24 (0.03–1.75) | 0.159 | 0.43 (0.06–3.26) | 0.411 |
| Reconstruction material |  |  |  |  |
| Breast prosthesis | Reference |  | Reference |  |
| Autologous tissue | 0.56 (0.36–0.88) | 0.012 | 0.55 (0.34–0.90) | 0.016 |
| pT stage ≥3 | 0.96 (0.60–1.54) | 0.870 | 1.03 (0.61–1.77) | 0.902 |
| Nodal metastasis |  |  |  |  |
| 0 | Reference |  | Reference |  |
| 1–3 | 2.88 (1.23–6.75) | 0.015 | 2.64 (1.04–6.66) | 0.040 |
| 4≤ | 3.94 (1.62–9.60) | 0.003 | 3.87 (1.38–10.90) | 0.010 |
| Neoadjuvant chemotherapy | 0.63 (0.40–1.01) | 0.056 | 0.87 (0.48–1.59) | 0.654 |
| Adjuvant chemotherapy | 1.46 (0.98–2.17) | 0.064 | 1.08 (0.65–1.80) | 0.760 |
| Postmastectomy radiation therapy | 1.30 (0.88–1.91) | 0.191 | 1.11 (0.66–1.85) | 0.703 |

CI, confidence interval; OR, odds ratio.
